# Prevalence and impact of chronic ankle instability in female sport: a cross-sectional study

**DOI:** 10.1186/s13102-025-01211-5

**Published:** 2025-07-08

**Authors:** Lauren Forsyth, Luke Donovan, Rhona Martin-Smith, Patrick L. Rowe

**Affiliations:** 1 Faculty of Biomedical Engineering, University of Strathclyde, Glasgow, UK; 2 Department of Applied Physiology, Health, and Clinical Sciences, University of North Carolina at Charlotte, Charlotte, USA; 3 College of Sport, Health & Engineering, Victoria University, Melbourne, Australia

**Keywords:** Chronic ankle instability, Lateral ankle sprain, Females in sport, Soccer, Netball, Volleyball, Basketball

## Abstract

**Background:**

The prevalence and impact of chronic ankle instability (CAI) is underreported among females participating in sports that are considered high risk for lateral ankle sprains. Identifying the prevalence and contextualising the impact may help reinforce the necessity of targeted rehabilitation and injury risk reduction strategies. The primary aim was to conduct an international study identifying the prevalence of CAI and impact on ankle function and quality of life of females participating in high-risk sports.

**Methods:**

The cross-sectional study across Australia, New Zealand, the United Kingdom (UK), and United States of America (USA), invited females (≥ 18 years old) participating in netball, soccer, basketball, or volleyball to complete an online survey about their ankle health. A convenience sample was recruited online via each of the researchers covering their respective region. The survey comprised questions related to general demographic/health information, and validated questionnaires—Cumberland Ankle Instability Tool (CAIT)/Foot and Ankle Ability Measure-Sport (FAAM-S)/Health-Related Quality of Life Score (HRQOL). Participants were classified with CAI if an ankle sprain and CAIT score of < 25 were recorded on the same ankle. A CAIT score of > 24 identified either a potential coper (someone reporting a previous ankle sprain) or healthy participant (no previous ankle sprains). An alpha level of *p* < 0.05 denoted statistical significance.

**Results:**

Five-hundred seventy-eight responses were received. Of those, 258 had complete datasets from across the UK (44%,*n* = 170), Australia (27%,*n* = 106), New Zealand (19%,*n* = 75), and USA (7%,*n* = 29). Ankle sprains were the most common ankle injury (77%). 73% reported CAI of their left ankle and 54% reported CAI of their right ankle. The FAAM-S (*p <* 0.05) and HRQOL (*p >* 0.05) scores were reduced for the CAI group, compared to both the potential copers and individuals with no ankle injury. When stratified by sport there were no differences across outcome measures.

**Conclusion:**

Chronic ankle instability is prevalent in females who participate in sports determined as high risk of ankle sprains. The results diversify knowledge of CAI in women across a broader participation level and distribution of sports than previously reported. Prevention strategies must be implemented globally to minimise the impact of CAI on performance and quality of life.

**Supplementary Information:**

The online version contains supplementary material available at 10.1186/s13102-025-01211-5.

## Key points

The results of this paper diversify our knowledge of CAI in females participating in sport across a wider participation level and distribution of sports than previously reported.

Chronic ankle instability is prevalent in females who participate in sport; they report high levels of instability and significantly decreased health-related quality of life. Females participating in sport were more likely to report instability of the ankle as opposed to a functional deficit.

**Supplementary Information:**

The online version contains supplementary material available at 10.1186/s13102-025-01211-5.

Lateral ankle sprains are the most common lower limb injury in sport [1, 2]. The risk of females sustaining an ankle sprain is approximately double that of males, and indoor/court sports with pivoting report the highest incidence of ankle sprains [1, 3]– i.e. Basketball, netball, soccer, and volleyball. Following an index ankle injury, individuals are at a six-fold increased risk of re-injury [2], including 20–75% developing chronic ankle instability in the general population (CAI) [4]. CAI is a multi-faceted clinical condition associated with recurrent ankle sprains; ongoing symptoms include pain, restricted arthrokinematics; mechanical laxity; perceived instability [4, 5, 6]. Concerningly, recurrent sprains are prevalent in sporting populations.

The prevalence of CAI is population-dependent, with lower prevalence in recreationally active individuals and highest prevalence in sports that frequently require various combinations of running, jumping, and cutting (i.e. soccer, basketball, dance) [7, 8]. Specifically, among the aforementioned sports, prevalence of CAI has been reported as high as 80% [9]- [10]. In addition to types of sports, prevalence of CAI appears also to be influenced by sex where CAI is greater among female athletes when compared to males [11]. Considering that CAI prevalence is influenced by both sport/activity and sex, there is a need for sport/sex-specific studies on the prevalence of CAI. However, current literature has not investigated female athletes in isolation, and have not included multiple levels of sport participation within their sample. Given those who participate in sport sustain ankle injuries at far higher rates than those who do not, we must further understand the potential long-term implications for females specifically participating in these high-risk sports—ie. netball, soccer, basketball, and volleyball across multiple levels of sport.

The long term physiological and psychological effects of CAI causes a substantial burden on an individual’s quality of life, including their ability to work and participate in physical activity [4]. Measures of health-related quality of life (HRQOL) have shown significant deficits in samples of 25 and 68 physically active people with CAI compared to healthy individuals [12, 13]. Ankle function is also significantly impacted in populations with CAI and can accurately be measured using the Foot and Ankle Ability Measure (FAAM)—specifically the sport-specific scale (FAAM-S) [12]—likely further impacting quality of life.

A catalyst to these negative outcomes is the link between CAI and ankle osteoarthritis [14], with 80% of cases diagnosed as post-traumatic [2, 15]). In addition to the link between CAI and ankle OA, sex and athletic history appear to be confounding variables where women and retired athletic populations have a greater prevalence of ankle OA [16, 17]. For example, retired soccer players who had sustained at least 1 ankle injury were 1.3 times more likely to have developed ankle OA [10]. As such, decreasing joint injuries (e.g. lateral ankle sprains and fractures), particularly for women in high risk sports, will likely sustainably reduce the incidence of ankle OA [18].

There are clear long term health consequences to an initial ankle injury, and the presence of CAI. Knowledge of the prevalence of these injuries is important to inform key stakeholders, identify prevention and rehabilitation strategies, and enhance resource prioritisation. To develop targeted rehabilitation and injury risk reduction strategies, defining the problem of CAI across various sporting groups is warranted. However, research identifying the prevalence of CAI to date has been limited to small sample population-based studies, mainly in high performing athletes, military personnel, and younger populations [7, 9, 11, 19, 20]. A systematic review by Lin et al. [9] reviewed the epidemiology of CAI with perceived ankle instability. Nine studies were included from France, USA, Japan, Ireland, and Australia [9]. An additional study examined CAI in Taiwanese basketball players [11]. This highlights a lack of published data internationally, but particularly across New Zealand and the United Kingdom. Furthermore, research on prevalence of CAI has pre-dominantly focused on young adult athletes, reporting on student cohorts with an average age of 22.4 ± 3.4 years [9, 11]. Of this research, none assessed solely females who participate in sport across various levels from social to elite [9], and none has compared the prevalence of CAI across regions. This is necessary to investigate intercountry differences in management practices and return to play decisions that have been reported in netball between Australia and the UK may result in differences between CAI prevalence [21].

The primary aim of this study was to conduct a large-scale international study to identify the prevalence of CAI and impact on ankle function and quality of life of females participating at multiple levels of high-risk sports such as netball, football, basketball, and volleyball. The study findings will report necessary sport/sex-specific data and provide clinicians with important information regarding the impact of CAI on females participating in high-risk sports.

## Methods

### Study design

The investigation was a cross-sectional study across Australia, New Zealand, the United Kingdom, and the United States of America. The study was approved by the University of Strathclyde ethics committee (DEC.BioMed.2024.365) and University of North Carolina at Charlotte Institution Review Board.

### Participants

The study was advertised online as an investigation of ankle health in female sport. Four sports were specifically targeted (netball, soccer, basketball, and volleyball) given the high risk of ankle injuries in each as previously reported [1, 7, 9]. Therefore, if females (aged 18 years old and over) played one of these sports, and had done so for at least 12 months (irrespective of frequency of participation—eg. one season and/or weekly practice), they were invited to complete the online survey. If women consented to the survey but did not identify as female, did not play one of these sports, or had not played the specified sport for at least 12 months they were automatically exited from the survey.

### Survey components

The international multidisciplinary research team (*n* = 4) developed the survey based on the CAI literature gaps identified which aimed to collect information on ankle and general health and wellbeing of athletes in high-risk sports (see Appendix A).

The survey comprised questions related to general demographic information, general health history, and validated patient-outcome questionnaires. This included 5 sections in total and was approved by the research team before distribution to participants. These are described below.

Section 1 consisted of questions regarding demographic information including participant’s age, sex, sport participation, and ankle injury history. To determine level of sport participation, the survey asked participants to define their (1) current and (2) highest level of play. The list differed whether participants reported being from the UK/Australia/New Zealand or the from the USA given the different structure of competition (Table 1).

**Table 1:** Different levels of play for different locations

Level of Play in UK, Australia, and New Zealand	Level of Play in USA
Professional	National
International	Professional
National	Collegiate—NCAA Division I
Club level	Collegiate—NCAA Division II
Recreational	Collegiate—NCAA Division III
Social	Collegiate—NAIA
	Collegiate—National Junior College Athletic Association
	University/College Club Level
	High School/Travel League
	Recreational/Social/Intramural

NCAA: National Collegiate Athletic Association; NAIA: National Association of Intercollegiate Athletics

Prior to completing the section on ankle injury history (Sect. 2) the following definitions were provided, given that the data collected was reliant on injury recall:

*Ankle sprains are the most common injury sustained by athletes and active individuals. In sports involving running*,* quick stops and starts*,* cutting*,* and changing directions ankle sprains often occur. This can be from the ankle rolling when weight is planted on the outer edge of the foot or when an individual lands on uneven ground or another player’s foot. There may be a popping sound when this happens as the ankle ligaments stretch/tear. The pain may be minimal and might quickly go away but in some cases the ankle may become swollen*,* painful to walk/run*,* and require medical treatment. In such cases crutches*,* walking boots*,* or braces may be prescribed for support and protection.*

*International ankle consortium definition of ankle sprain:*

An acute traumatic injury to the lateral ligament complex of the ankle joint as a result of excessive inversion of the rear foot or a combined plantar flexion and adduction of the foot. This usually results in some initial deficits of function and disability.

*International ankle consortium definition of giving way:*

*“The regular occurrence of uncontrolled and unpredictable episodes of excessive inversion of the rearfoot (usually experienced during initial contact during walking or running)*,* which do not result in an acute lateral ankle sprain.”*

Participants were asked to specify also if previous ankle injuries included fractures. The International Ankle Consortium (IAC) criteria for CAI excludes those with a history of ankle fractures as alterations (both neuromuscular and structural) to the ankle may confound results relating to instability [5]. Koshino et al. [22] previously reported that the prevalence of CAI in the studied population doubled when the IAC exclusion criteria was not taken into consideration as well as the inclusion criteria for CAI.

Following participant demographic information, participants completed three validated questionnaires assessing ankle function (Sect. 3), instability (Sect. 4), and health-related quality of life (Sect. 5).

The Cumberland Ankle Instability Tool (CAIT) is a recognised valid and reliable tool for assessing CAI stability (intraclass correlation coefficient [ICC]_2,1_ = 0.96) [23]. The 9-item questionnaire has a maximum score of 30, and a lower score indicates a lower perception of ankle stability. According to the International Ankle Consortium, a score of < 25 indicates CAI [24].

The Foot and Ankle Ability Measure (FAAM) was used to assess foot and ankle function and is a recognised tool for assessing CAI by the International Ankle Consortium [24, 25, 26]. Specifically, the survey included the FAAM-Sport (FAAM-S) which consists of 7 items measuring ability to performing sporting-related tasks such as running, jumping, and pivoting (intraclass correlation coefficient [ICC]_2,1_ = 0.87) [27]. Scores were counted from 0 to 32 and translated to a percentage of the total possible score. A lower score indicated lower function. According to the International Ankle Consortium (IAC), a score of < 80% for the FAAM-S subscale may indicate CAI [24]. It should be noted that the FAAM-S threshold from the IAC is used as description of impairment, whereas the CAIT threshold of < 25 is used as diagnostic criteria for CAI [24].

The HRQOL-14 questionnaire has been reported in CAI literature [13, 28]. The questionnaire consists of 14 items which includes a mixture of multiple choice and text answers relating to athlete health over the past 30 days. A lower score indicates a lower reported quality of life. There are currently no reported cut off scores for quality of life in relation to CAI.

### Survey distribution

The survey was distributed internationally to collect a convenience sample (United Kingdom, USA, Australia, & New Zealand) for a period of 6 weeks (9th March 2024 to 22nd April 2024) via the web-based platform Qualtrics (Qualtrics, Provo, UT). The survey took approximately 20–25 min for a participant to complete. Given the survey was online, an online recruitment strategy was appropriate. The advertisements were shared across social media (X (formerly twitter), Facebook, and LinkedIn) and emailed directly to sports clubs, physios, athletic trainers, and sports coaches, strength and conditioning coaches, sport scientists, and athletes to share amongst their networks. On Facebook, groups were targeted for all sports such as ‘Netball Coaches Corner’ and ‘Scottish women’s recreational football’ where the groups consisted of thousands of women involved in the sports. To reduce selection bias, survey advertisements were clearly stated that any female participating in any one of these sports could complete the questionnaire, regardless of whether they had sustained any ankle injuries.

### Data analysis

Participants were classified as either ankle sprain, ankle fracture, or no ankle injury. The CAIT and FAAM-S scores were split into groups according to their scores—CAIT: <25 or ≥ 25; FAAM-S: <80% or ≥ 80%. These cut offs were to support the International Ankle Consortium diagnostic criteria reported by Gribble and colleagues [5].

To categorise those with CAI, each participant’s CAIT scores were used only. If participants had had a previous ankle sprain and reported a score of < 25 on the CAIT they were recorded as having CAI [5]. Please note that our definition does not fully adhere to Gribble and colleagues’ [24], however a clear classification is shown in Table 2.

Participants who reported a previous ankle sprain but no CAI were categorised as ‘potential copers’. This group could not be definitively classified as Copers since the minimum recording standards for a Coper group were not met (i.e. time since last sprain was not collected in the survey) [29].

Participant’s ankles were analysed independently, thus each CAIT, FAAM-S, and HRQOL score was analysed for each ankle. Table 2 details how participants were classified into groups—CAI, Potential Coper, No Ankle Injury, and Not Applicable (n/a). If participants were not applicable to any group they were not included in the specific analysis.

**Table 2:** Classification of groups for analysis

	CAIT < 25 (left)	CAIT > 24 (left)	CAIT < 25 (right)	CAIT > 24 (right)
Ankle Sprain (left)	CAI	Potential Coper	n/a	n/a
Ankle Sprain (right)	n/a	n/a	CAI	Potential Coper
No Ankle Sprain (left)	n/a	No Ankle Injury	n/a	n/a
No Ankle Sprain (right)	n/a	n/a	n/a	No Ankle Injury

CAIT: Chronic Ankle Instability Tool

Partial responses (> 13% of the survey completed) were included in the final analysis.

### Statistical analysis

All statistical testing was conducted using Statistical Package for Social Sciences software (SPSS Statistics: v. 26, IBM, USA). Participant demographics were summarised by means ± SDs for non-categorical data.

Non-parametric tests analysed the questionnaire data because of ceiling effects of these measures. Statistical differences between the groups were assessed using the nonparametric Kruskal-Wallis test for CAIT and FAAM-S, and parametric ANOVA for HRQOL. Chi-square tests analysed the relationship between categorical data. Simple linear regressions were used to analyse the relationship between predictor values (e.g. CAIT score and ankle injury on HRQOL score). An alpha level of (*p* < 0.05) was set to denote statistical significance.

## Results

**Fig. 1:**
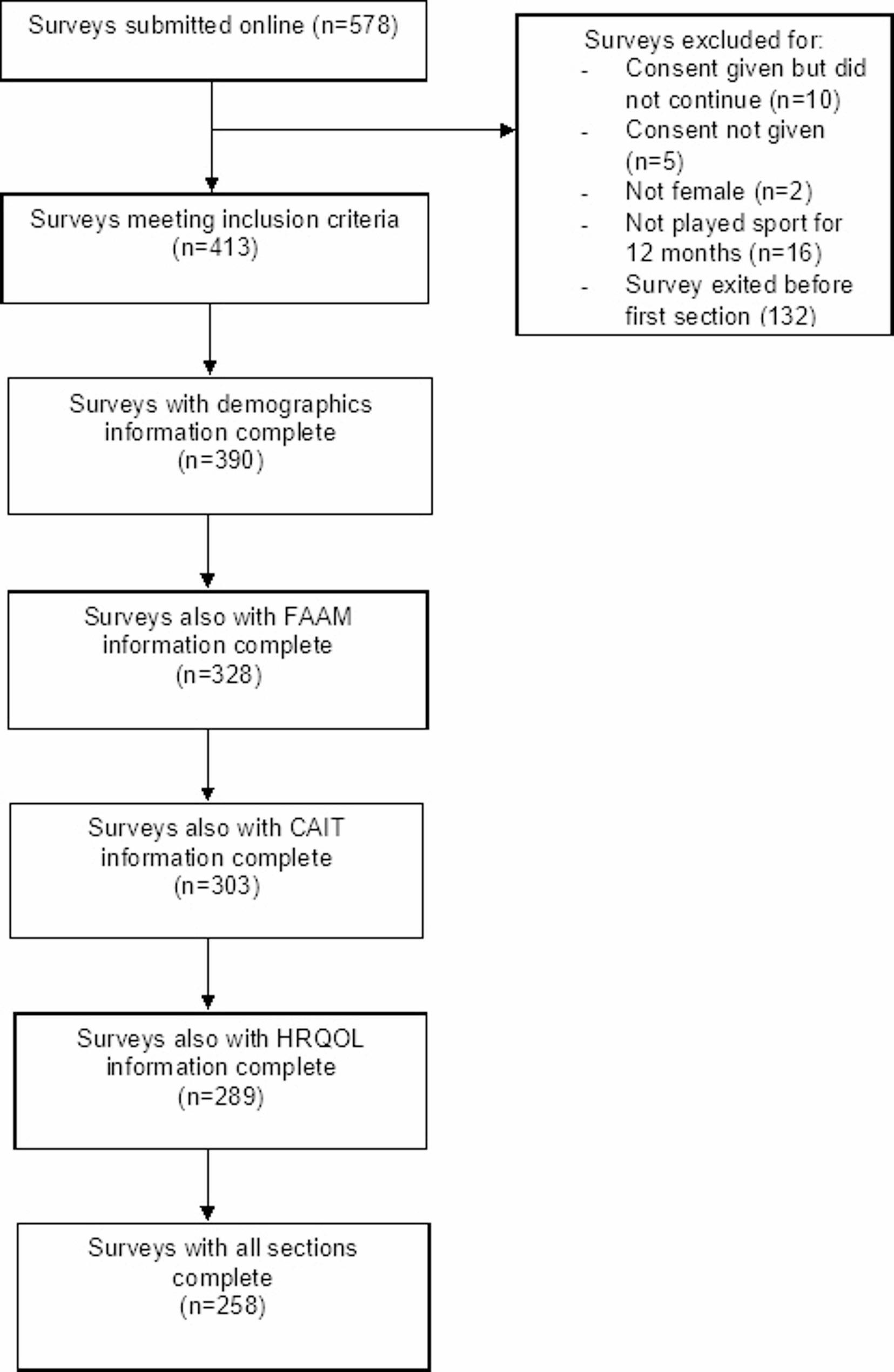
Participant flow diagram

A total of 578 responses were submitted online. A breakdown of the final number of surveys that were returned completed is shown in Fig. 1. One hundred and seventy-five participants did not meet the inclusion criteria or did not proceed after giving consent, leaving 413 participants continuing to the first section of questions on participant demographics. Of those 413, 62.5% (258/413) continued to complete all sections of the survey.

Of the 390 participants who completed the demographic data section of the survey (Fig. 1), netball was the most participated sport (73.7%) followed by soccer (15.3%). The location of participants was mainly split across the UK (43.5%) and Australia (27.1%) (Table 3).

Participants’ ages were primarily distributed across the 18–24 (32.2%), 25–29 (20.7%), and 30–39 (28.9%) categories (Table 3). Of the remaining 17.9% in the 40 + category, 12% were 40–49, 4.1% were 50–59, 1.3% were 60–69, and 0.3% were 70–79 and 90+. Although mass varied among the different sports, the average height was very similar for each sport ranging between 1.69 and 1.73 m.

The highest level of sport played across the UK, Australia, and New Zealand was professional/international (1.1%) and the lowest was recreational/social (23.6%). The most common level of sport played was reported as club level (72.4%). In the USA, given the different play structure than the other locations, the most prevalent level of play was College NCAA Division I (75%).

**Table 3:** Descriptive statistics for CAI, coper, and no ankle injury participants. Results are presented as number for dichotomous data– n (%)– and mean (SD) for continuous data

	All	Netball	Soccer	Basketball	Volleyball
**Number of** **participants**	390	288 (73.7%)	60 (15.3%)	13 (3.3%)	29 (7.4%)
**Age**					
18–24	126 (32.2%)	91 (27.4%)	28 (46.7%)	6 (46.2%)	13 (44.8%)
25–29	81 (20.7%)	61 (21.2%)	11 (18.3%)	2 (15.4%)	7 (24.1%)
30–39	113 (28.9%)	83 (28.8%)	17 (28.3%)	4 (30.8%)	9 (31%)
40+	70 (17.9%)	65 (22.6%)	4 (6.7%)	1 (7.7%)	0
**Country**					
Australia	106 (27.1%)	91 (31.6%)	7 (11.7%)	4 (30.8%)	4 (13.8%)
UK	170 (43.5%)	125 (43.3%)	27 (45%)	6 (46.2%)	12 (41.4%)
USA	29 (7.4%)	0	19 (31.7%)	1 (7.7%)	9 (31%)
NZ	75 (19.2%)	65 (22.6%)	6 (10%)	2 (15.4%)	2 (6.9%)
Other	10 (2.6%)	7 (2.4%)	1 (1.7%)	0	2 (6.9%)
**Time Played** **Sport (years)**	16.66 (8.91)	17.03 (9.40)	15.17 (7.28)	17.08 (8.42)	12.28 (6.97)
**Mass (kg)**	73.66 (15.36)	74.37 (15.07)	70.43 (15.50)	77.17 (23.22)	71.79 (13.16)
**Height (m)**	1.70 (0.08)	1.70 (0.08)	1.69 (0.07)	1.72 (0.10)	1.73 (0.07)

Of 383 female athletes who recorded their ankle injury in the survey, 77% had sustained an ankle sprain to either the left ankle, right ankle, or both ankles. This distribution was representative across all sports (Table 4). Most athletes (47.7%) had sprained both ankles, compared to only right (18.8%) or only left (10.4%). Netball and volleyball were the sports with the highest prevalence of ankle sprains in the population samples.

**Table 4:** Descriptive statistics for ankle injuries and the number of ankle sprains. Results are presented as number for dichotomous data– n (%)– and mean (SD) for continuous data

	All (*n* = 383)	Netball	Soccer	Basketball	Volleyball
**Ankle injury**					
Sprain	295 (77.0%)	226 (79.0%)	39 (68.4%)	8 (61.5%)	22 (81.5%)
Fracture	40 (10.4%)	30 (10.5%)	8 (14.0%)	2 (15.4%)	0
No ankle injury	48 (12.5%)	30 (10.5%)	10 (17.5%)	3 (23.1%)	5 (18.5%)
**Average number of sprains**					
Right	3.47 (5.88)	3.41 (5.69)	3.22 (6.08)	3.23 (3.32)	4.69 (7.98)
Left	2.81 (5.37)	2.81 (5.13)	2.57 (5.92)	3.31 (6.69)	3.28 (6.09)

### Cumberland ankle instability tool

**Fig. 2:**
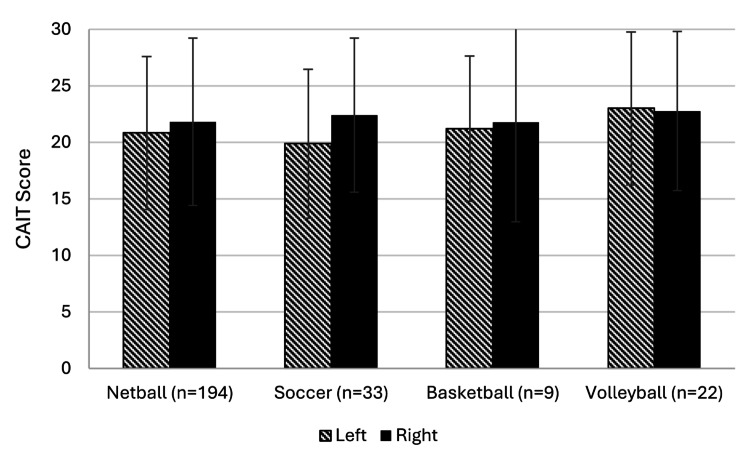
Mean (SD error bars) of the CAIT for each sport

Across all sports the CAIT scores were similar, with no significant interactions between sports and between the right and left side (*p* > 0.05) (Fig. 2). Overall, all sports reported a CAIT score below the CAI threshold (< 25). Soccer recorded the lowest score on the left side, while basketball recorded the lowest score on the right side. The greatest variation of CAIT scores within sport was in netball for the left side, and basketball for the right side.

As the number of sprains increased, the CAIT score decreased. The number of sprains significantly predicted the CAIT score for both the left side*—*F(1,218) = 11.98, *p* < 0.001, R^2^ = 0.05*—*and the right side*—*F(1,255) = 10.52, *p* < 0.001, R^2^ = 0.04.

**Table 5:** Mean (SD) for questionnaire data recording instability, function, and health0related quality of life

	All (*n* = 258)	Ankle Sprain (right) (*n* = 186)	Ankle Sprain (left) (*n* = 159)	Ankle Fracture (*n* = 29)	No Ankle Injury (*n* = 24)
**CAIT L** ^*al ar b*^	20.91 (6.73)	19.53 (6.69)	20.40 (6.62)	20.97 (5.31)	27.25 (3.29)
**CAIT R** ^*al c*^	21.98 (7.30)	21.73 (7.3)	20.05 (7.16)	21.83 (7.83)	26.25 (5.36)
**FAAM**-S ^*al*^	80.06 (22.92)	79.58 (21.87)	78.35 (23.22)	76.14 (23.75)	92.90 (20.98)
**FAAM**-S **Q10. (Rate function out of 100)**^*b*^	80.33 (23.07)	79.20 (22.79)	79.09 (22.79)	79.28 (20.64)	91.50 (23.86)
**HRQOL score**	16.86 (2.06)	16.77 (2.07)	16.74 (2.00)	16.83 (1.98)	17.42 (2.30)

CAIT: Chronic Ankle Instability Tool; FAAM-S: Foot & Ankle Ability Measurement-Sport; Q10: Question 10; HRQOL: Health-Related Quality of Life Score

^*al*^significant difference between ankle sprain left and no ankle injury (*p <* 0.05)

^*ar*^significant difference between ankle sprain right and no ankle injury (*p <* 0.05)

^*b*^significant difference between ankle fracture and no ankle injury (*p <* 0.05)

^*c*^significant difference between ankle sprain and ankle fracture (*p <* 0.05)

**Fig. 3:**
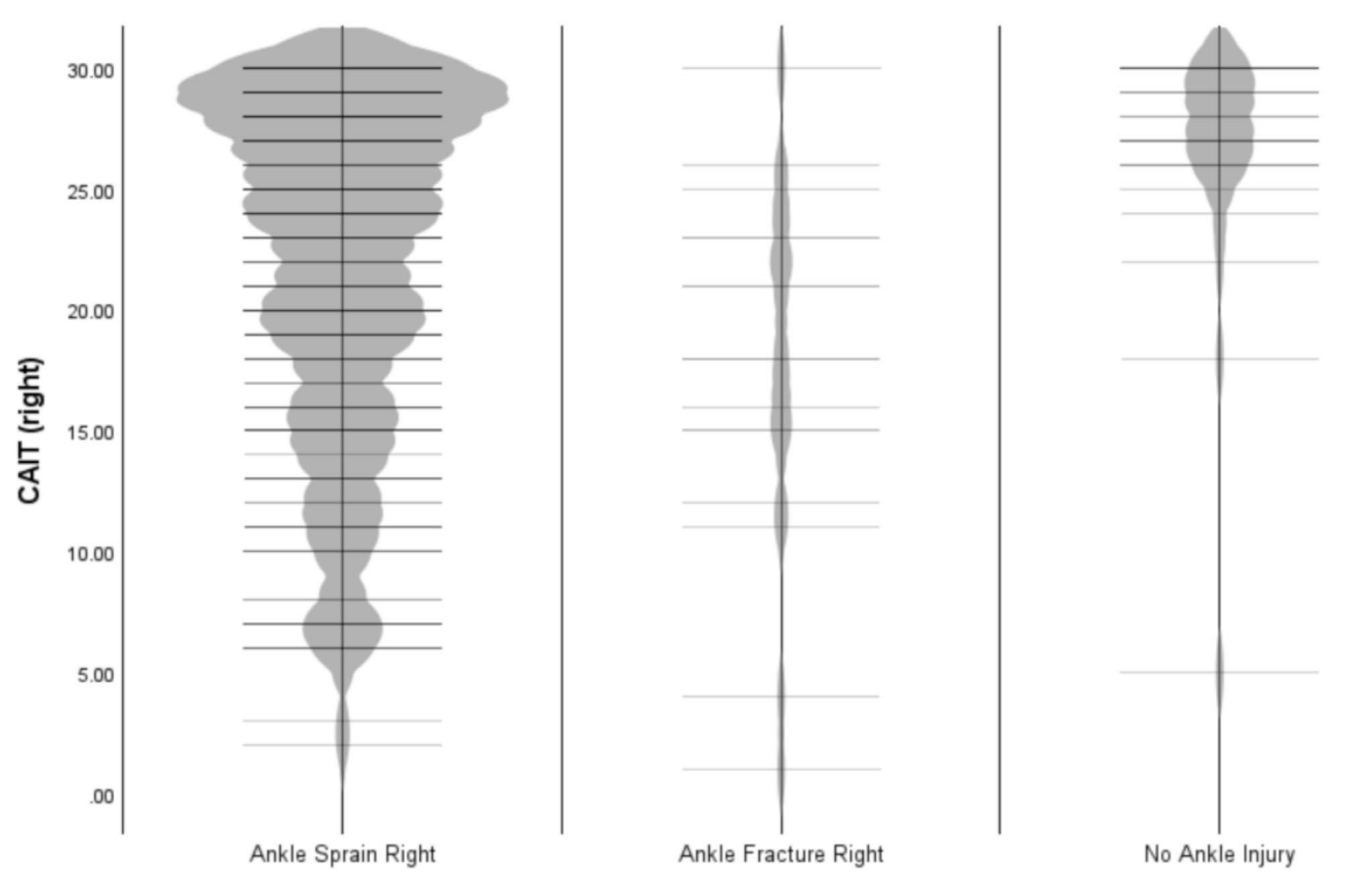
Violin plot to show CAIT score distribution across three groups for the right ankle

**Fig. 4:**
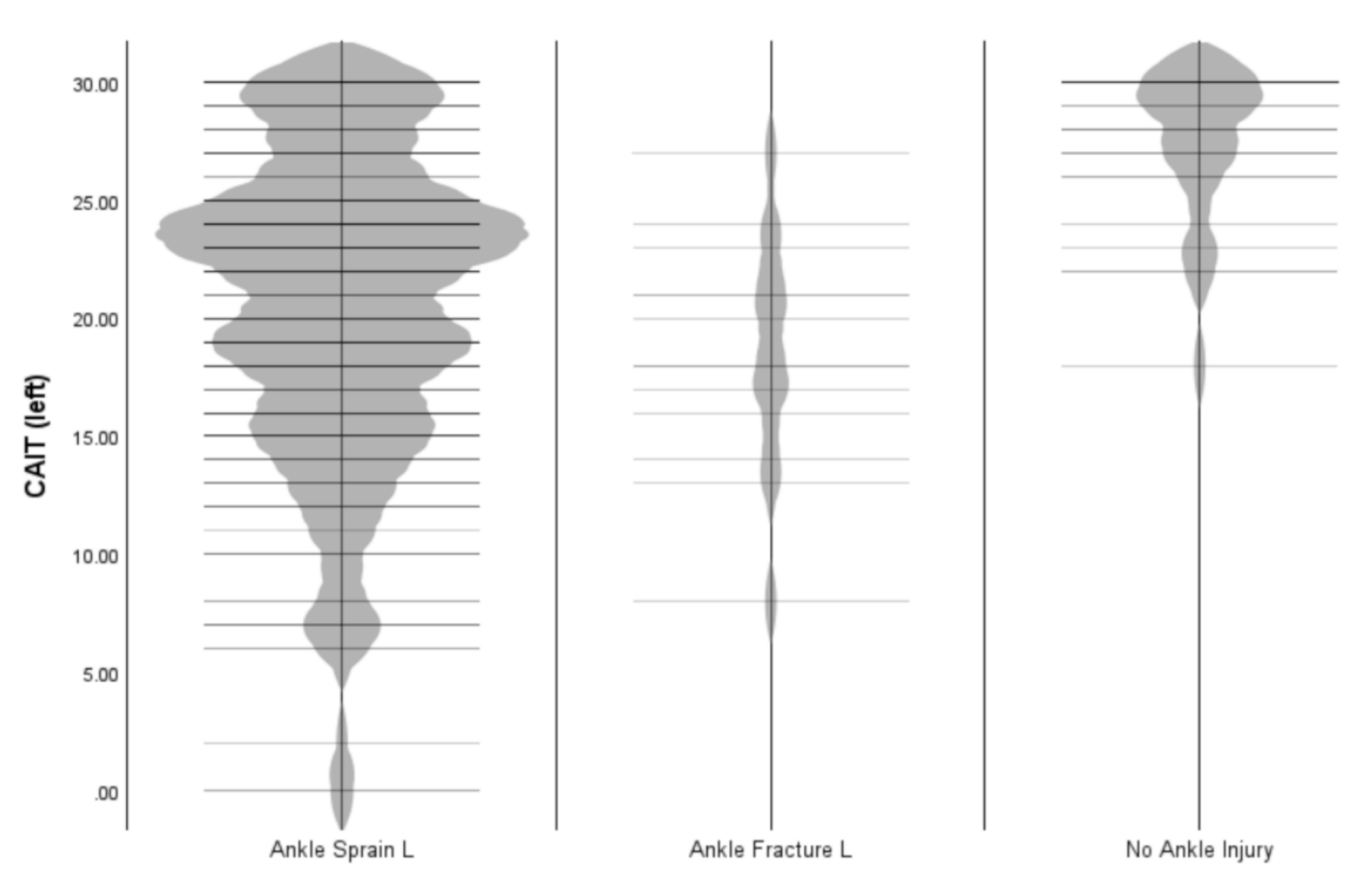
Violin plot to show CAIT score distribution across three groups for the left ankle

The average CAIT scores for each injury group are reported in Table 5, and the distribution is shown in Figs. 3 and 4. The groups included 186/258 individuals reporting a right ankle sprain (72.1%), 16/258 reporting a right ankle fracture (6.2%), and 24/258 reporting no ankle injury (9.3%). For the left ankle the groups included 159/258 individuals reporting a left ankle sprain (81.8%), 13/258 reporting a left ankle fracture (6.6%), and 24/258 reporting no ankle injury (9.3%).

The CAIT score was significantly lower for the left side only in the ankle sprain group compared to those with no previous ankle injuries (Table 5). From the CAIT data 199/258 participants (77.1%) were below the CAI threshold (< 25) for CAI classification. This included 173/258 (67.1%) cases of left CAI and 134/258 (51.9%) cases of right CAI. Further analysis was conducted to determine whether CAI classification was consistent with the ankle which had sustained a sprain. Of the 159 who had sustained a previous left ankle sprain, 116 (73.0%) also reported CAIT < 25 on the left side and thus were classified as having CAI of the left ankle. Of the 186 who had sustained a previous right ankle sprain, 101 (54.3%) also reported CAIT < 25 on the left side and were classified as having CAI of the right side. This resulted in unilateral CAI in 57 athletes (22.1%) and bilateral CAI in 80 athletes (31.0%).The Potential Coper groups (previous ankle sprain but no CAI according to the CAIT threshold) consisted of 43/159 (27.0%) athletes for the left side and 85/186 (45.7%) athletes for the right. As indicated above this was categorised from athletes suffering a previous ankle sprain but no CAI according to the CAIT threshold in that ankle. CAIT scores were significantly less in the CAI group compared to the Potential Copers (Table 6).

**Table 6:** Categorised data of those with CAI and resulting CAIT scores. The potential copers group had sustained an ankle sprain but did not record a CAIT score of < 25. Results are presented as mean and standard deviation

CAIT			95% Confidence Intervals	
	Mean	SD	Lower bound	Upper bound
**CAI (left) (***n* = 116)^†^*	17.71	5.63	17.08	20.28
**CAI (right) (***n* = 101)^†^*	16.48	5.76	15.11	18.31
**Potential Copers (left) (***n* = 43)^†^	27.67	1.20	26.073	29.270
**Potential Copers (right) (***n* = 85)^†^	27.98	1.75	26.74	29.13
**No Ankle Injury (left) (***n* = 9)*	28.33	1.94	24.77	31.90
**No Ankle Injury (right) (***n* = 8)*	28.00	2.20	24.32	31.68

CAIT maximum attainable score is 30. The upper bound of the 95% confidence interval may exceed this

^†^significant difference between CAI and Potential Copers (*p <* 0.05)

^*^significant difference between CAI and No Ankle Injury (*p <* 0.05)

**Fig. 5:**
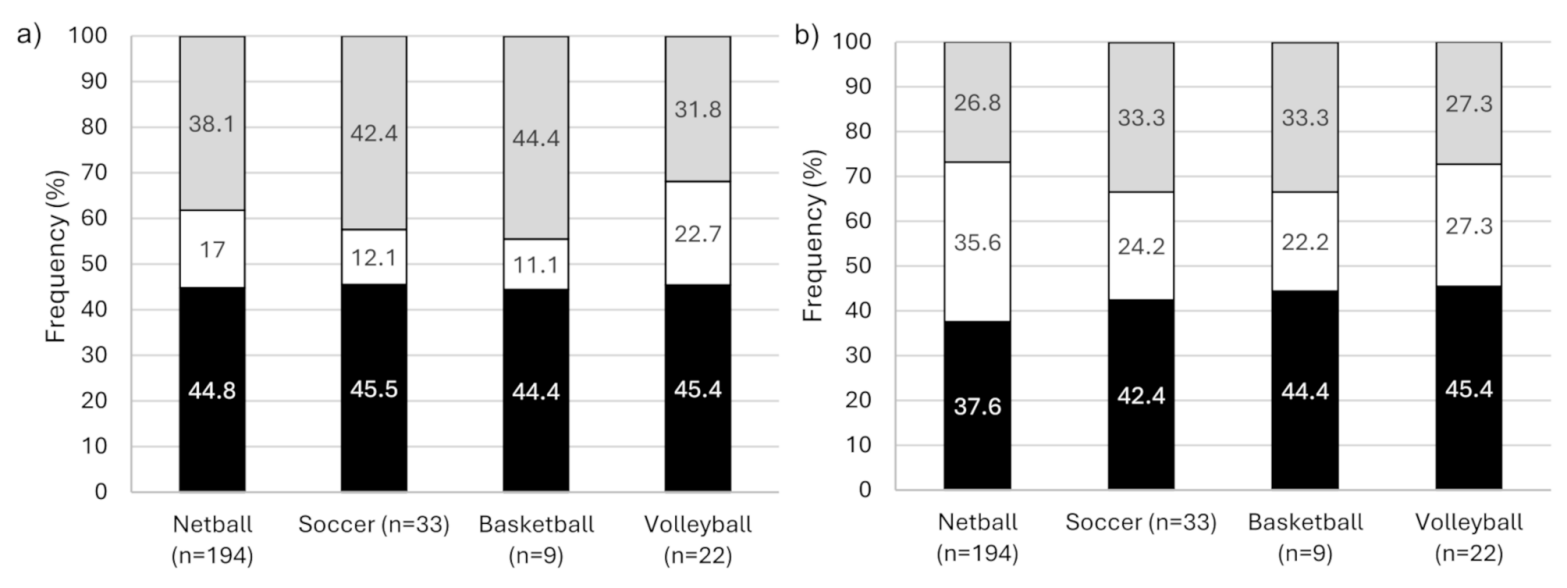
Distribution of CAI across the sports for **(a)** the left side and **(b)** the right side. Black shading: Ankle Sprain + CAI; White shading: Ankle Sprain + No CAI; Grey shading: Remainder of population

When stratified by sport, all sports reported a prevalence of CAI between 37.6 and 45.5% (Fig. 5). The average CAIT scores for those with CAI across the sports ranged from 17.13 to 18 (left) and from 16.10 to 21 (right). CAIT scores significantly differed within the sport between the CAI and Potential Coper groups across all sports (*p* < 0.05), but there were no significant interactions between the CAIT scores across the different sports (*p* > 0.05).

### Foot and ankle ability measure-sport

The lowest score on ankle function using the FAAM-S was reported in the ankle fracture group (Table 5). However the lowest score on the question to rate function out of 100 was lowest for the ankle sprain group (left ankle). There were no significant differences in FAAM-Sport scores between the sprain and fracture groups, but both did differ significantly to the non-injured group (*p* < 0.05). As the number of sprains increased, the FAAM-S score decreased, but the total number of sprains did not significantly predict the FAAM-S score—F(1,210) = 2.13, *p* < 0.146, R^2^ = 0.01.

From the FAAM-S data 93/258 (36%) were below the FAAM-S < 80% threshold. This was less than half of the number who reported low CAIT scores in the < 25 threshold, and therefore have developed perceived instability. Of those 79/93 (85%) had sustained at least 1 ankle sprain on either their left or right ankle, or both. This was compared to 126 who did have previous ankle sprains but still reported high function considering the FAAM-S threshold.

The final question of the FAAM-S rates overall function. Those with ankle sprains (205) reported most often having abnormal (n, %) (85, 41.5%) and severely abnormal (87, 42.4%) function. As did those with fractures (30) reported abnormal (15, 51.7%) and severely abnormal (10, 34.5%) function. The group with no previous ankle injuries also reported having severely abnormal function in 83.3% (20/24) of cases.

When categorising data for those with CAI (according to the CAIT score cut off of < 25) 49.1% (left) and 53.5% (right) of athletes with CAI were below the FAAM-S threshold (< 80). The FAAM-S scores were significantly reduced for the CAI group, compared to both the potential copers (those with a previous ankle sprain and CAIT score > 24) and No Ankle Injury individuals (Table 7). When stratified by sport, there were no significant interactions between the FAAM-S scores of the different sports in those with CAI (*p* > 0.05).

**Table 7:** Categorised data of those with CAI and resulting FAAM total scores. The potential copers group had sustained an ankle sprain but did not record a CAIT score of < 25. Results are presented as mean and standard deviation

FAAM-S			95% Confidence Intervals	
	Mean	SD	Lower bound	Upper bound
**CAI (left) (***n* = 112)^†^*	75.37	21.98	63.92	77.41
**CAI (right) (***n* = 98)^†^*	74.24	20.39	67.25	82.22
**Potential Copers (left) (***n* = 43)^†^	86.12	24.80	78.58	92.02
**Potential Copers (right) (***n* = 83)^†^	85.88	21.99	82.02	92.13
**No Ankle Injury (left) (***n* = 7)*	99.11	1.52	82.41	115.81
**No Ankle Injury (right) (***n* = 7)*	99.55	1.18	83.06	116.05

FAAM maximum attainable score is 100. The upper bound of the 95% confidence interval may exceed this

^†^significant difference between CAI and Potential Copers (*p <* 0.05)

^*^significant difference between CAI and No Ankle Injury (*p <* 0.05)

### Health-related quality of life

HRQOL scores were lowest in the ankle sprain group (*p >* 0.05) (Table 5). As the number of sprains increased, the HRQOL score decreased, but the total number of sprains did not significantly predict the HRQOL score*—*F(1,217) = 1.75, *p* < 0.01, R^2^ = 0.01.

As CAIT score (both left and right) decreased, the HRQOL score decreased, and significantly predicted the HRQOL score—F(2,256) = 11.70, *p* < 0.00, R^2^ = 0.084. Upon further analysis of those with previous ankle injuries and a CAIT score of below 25 (indicating CAI), those with CAI reported lower HRQOL scores than those without (*p >* 0.05) (Table 8). When stratified by sport, there were no significant interactions between the HRQOL scores of the different sports in those with CAI (*p* > 0.05).

**Table 8:** Categorised data of those with CAI and resulting HRQOL scores. The potential copers group had sustained an ankle sprain but did not record a CAIT score of < 25. Results are presented as mean and standard deviation

HRQOL			95% Confidence Intervals	
	Mean	SD	Lower bound	Upper bound
**CAI (left) (***n* = 116)	16.53	2.00	15.94	17.18
**CAI (right) (***n* = 101)	16.42	1.86	15.85	17.10
**Potential Copers (left) (***n* = 43)	17.30	1.91	16.63	17.88
**Potential Copers (right) (***n* = 85)	17.20	2.24	16.82	17.75
**No Ankle Injury (left) (***n* = 9)	17.78	1.56	16.43	19.13
**No Ankle Injury (right) (***n* = 8)	17.75	2.05	16.33	19.18

HRQOL maximum attainable score is 25. The upper bound of the 95% confidence interval may exceed this

## Discussion

The primary aim of this study was to conduct an international study to identify the prevalence of CAI and quantify the impact of the condition on ankle function and quality of life of females participating in high-risk sports (netball, soccer, basketball, and volleyball). The study specifically focused on establishing the prevalence of CAI, as opposed to identifying risk factors. In summary, we found that CAI was highly prevalent among our participants and the condition was associated with a high-level of perceived instability and decreased HRQOL.

### Ankle injury prevalence

This study found that 88% of participants had sustained an ankle injury (sprain or fracture). Of those ankle injuries, sprains (77%) were much more common than fractures (10.4%). Among individuals who reported previous ankle sprains, 62% reported spraining both their left and right ankle. Our findings were somewhat expected given the results of previous epidemiological studies that also observed ankle sprains being highly prevalence among physically active individuals [2, 30, 31]. A systematic review by Fong and colleagues [32] further supports our findings as the review observed that among all ankle injuries sustained among volleyball, basketball, netball, and soccer athletes, ankle sprains accounted for 99.3%, 91%, 85.9%, and 76.8%, respectively.

### Ankle sprain prevalence across high-risk sports

Herzog et al. [1] recommended diversification in knowledge of ankle sprains across a wider participation level and distribution of sports than is currently available. Our study meets this challenge and supports previous findings that indoor court sports participants report the highest prevalence of ankle sprains, closely followed by soccer which was the greatest of the field sports [3, 33, 34].

Compared to other sports, Roos et al. [34] and Kerr et al. [33] both reported women’s volleyball, basketball, and soccer to have the highest number of ankle sprains. Neither of these studies included a netball population, and both sampled youth and collegiate athletes only. In netball populations, the ankle is injured more frequently than any other body region [35, 36]. A cohort study [36] that spanned three seasons found that 63.2% of netballers experienced a lateral ankle sprain. Although the specific prevalence of ankle sprains slightly differed across the studies, collectively, it is clear that volleyball, basketball, soccer, and netball are sports associated with a high prevalence of ankle sprains; thus this highlights the importance of integrating injury risk reduction programs among these sporting populations [21, 37].

### Prevalence of CAI

The prevalence of CAI was 73.0% for the left side and 54.3% for the right side. Research reports a wide range for CAI prevalence which is indicative of heterogeneity between studies [9, 38]. This may reflect the current CAI model described by Hertel and Corbett [6] which describes the condition as a spectrum with a range of symptoms and severity of symptoms. Lin et al.’s [9] systematic review summarised a CAI (and previous ankle sprain) prevalence of 46% (ranging from 9 to 76%). The results of our group are similar to those on the right side to Lin et al.’s review [9], with the left side at the upper end of this range. However, were greater than the prevalence reported by Kobayashi and colleagues [39] (9.4% CAI in those meeting IAC criteria), Tanen and colleagues [38](30.9% CAI in those with a previous ankle sprain), and Doherty et al. [3] (40% prevalence 1-year following lateral ankle sprain). Distribution of CAIT scores in this study also varied within the ankle sprain group. This highlights the heterogenous nature within the population itself and may be facilitated by the current CAI model described by Hertel and Corbett [6] and the inclusion criteria from Gribble and colleagues [24].

The interlimb difference of CAI prevalence is interesting, with CAI in the left limb recorded 18% more often. We did not collect dominate limb, however given that previous research has reported a six-fold increased risk of developing CAI 6-months post lateral ankle sprain when the non-dominant limb was sprained [39], future research should investigate.

When stratified by sport, our results were similar to the review by Lin and colleagues [9]. The female athlete population here reported 37.6–45.5% prevalence of CAI-, compared to 20–64% [9]. The highest prevalence of CAI was in netballers. These results reported 20% fewer cases of CAI compared to Attenborough et al. [40], likely due to their youth sample population who are known to record a higher prevalence of CAI [9], as well as the smaller number sampled. There are no reports on the prevalence of CAI in netballers in an older population like in the current study. For the remaining three sports (basketball, soccer, and volleyball) prevalence of CAI was greater compared to Tanen et al. [38], but similar to the more recent report by Koshino et al. [22]. Both studies recruited athletes from a variety of sports, however female inclusion was limited to 42.3% of 512 high school/college athletes [38] and 15.3% of 470 college athletes [22]. Koshino et al. [22] found that athletes who play basketball (63%, 14/22), volleyball (42%, 11/26) or soccer (37%, 15/40) had a high rate of CAI. The prevalence for CAI was lower in Tanen et al.’s study [38]12.2% in soccer (5/41), 25% in volleyball (9/36), and 29.8% in basketball (17/57). On analysis of the CAIT scores to suggest the severity of ankle instability in these groups, there is little research for comparison. The CAI group recorded lower severity scoring to the previous literature (17.71/16.48 vs. 19.64–21.2) [20]- [22, 41] suggesting that the female athletic population here have more severe CAI than what has been previously reported. However, we recognise that participants in this study may not extend to all individuals in the target population.

### Consequences of CAI on perceived function and health-related quality of life

Of those classified as having CAI, just over half of participants (51.3%) reported meeting the functional threshold (score < 80%, as per the FAAM-S). Previous research has also reported that female collegiate athletes with a previous ankle sprain were more likely to perceive a lower instability score than a lower disability score [42]. This suggested that although athletes were aware of their instability it did not appear to have affected perceived sporting performance to such a great degree. Furthermore, we believe that female athletes with significant functional disability following an ankle sprain may not have been captured in the study because they had already stopped their sports participation. This is hypothesised following reduced general physical activity levels in a population with CAI [43, 44]. The FAAM-S score did not significantly decrease as the number of sprains increased, but did significantly decrease when CAI (CAIT < 25) was present which is supportive of the previous literature in smaller sample populations [45]. Further information regarding how individuals may be adapting their daily function and tasks to manage their feelings of disability would be important to understand for further interpretation.

Those with CAI have typically reported a reduced HRQOL in previous literature [12, 28, 46]. Our results report a negative relationship between CAI and HRQOL in the left side only, and identified that a lower CAIT score overall was associated with a lower quality of life. Specifically, depression and ability to participate in social roles and activities have been identified to significantly negatively affect quality of life [28]. This is to be expected if individuals are forced to modify and/or limit daily activity and sports, particularly in a sporting population [4]. However, it is important to recognise that the present study may not have captured the true extent of this impact since athletes had to be currently participating in sport to complete the survey. Therefore, those with CAI who have since stopped sport participation, thus potentially impacting quality of life, will not have been included.

### Strengths and limitations

The main strengths of this study were the collaboration of researchers with expertise in CAI and high-risk sports, who then facilitated a large sample population that represented a diverse group of both the general and athletic population of all ages internationally. This is compared to most CAI research in smaller numbers of youth or college/university athletes without additional measures including ankle disability and HRQOL. In the current study there was a more representative distribution across all age ranges and additionally within the club level population which provides clinicians greater insight into the impact of CAI on these athletes. It would be of interest in future research to analyse the impact of age regarding CAI prevalence, level of disability, and HRQOL in a larger sample population. This study also stratified females in sport who have sustained ankle fractures and no previous ankle injuries. Although the number of participants was a strength of the study, our sample size was not evenly distributed across all variables, which could have introduced a risk of Type II error for secondary analyses that aimed to compare populations across sporting-levels or type of sport participation. With that said, the mean differences between groupings within these analyses were similar and did not exceed what would consistent a clinically meaningful difference.

The survey itself was likely subject to selection and recall bias. Although the survey was promoted as an ankle health survey for female athletespeople may have been more inclined to complete it if they had had an ankle injury, despite the wording being used to try and reduce the selection bias. Recall bias may have been present as people have to recall the injuries they have. Given the perception of ankle sprains in the general population as a benign injury there may have been discrepancies among individuals as to how to classify a sprain. An attempt to reduce this was made by defining ankle sprains in the survey before these questions were answered. Many different approaches were utilised to recruit participants online (e.g. email, social media). The route to survey completion was not recorded for participants thus we are unable to report which strategies led to the most success (i.e. greater enrolment). Therefore, our recommendation for future research is to report these findings to inform further research utilising an online approach.

Data collection was limited by not classifying whether the initial ankle sprain occurred at least 12 months prior to study enrolment and that the most recent injury must have occurred more than 3 months prior to study enrolment. These are two parts of the IAC standard inclusion criteria, thus participants classified as having CAI in this study may not have been classified with this further information [24]. In addition, participants were not asked to identify any other major injuries (e.g. other foot and ankle injuries such as syndesmosis/deltoid; meniscus tears, or anterior cruciate ligament injuries). Aside from this, all other criteria from the IAC inclusion and exclusions were met.

## Conclusion

The findings from this study highlight that CAI is prevalent across a diverse population of females participating in netball, basketball, soccer, and volleyball. The women were more likely to report an instability of the ankle opposed to a functional deficit, and the data suggests that those with CAI are negatively impacted reporting a decreased quality of life. Clinicians must acknowledge the prevalence of CAI, particularly feelings of instability and the negative impact on quality of life, and prevention strategies must be implemented globally to minimise the impact of CAI on sporting performance and quality of life.

## Electronic supplementary material

Below is the link to the electronic supplementary material.

Supplementary Material 1

## Data Availability

The datasets used and/or analysed during the current study are available from the corresponding author on reasonable request.

## Abbreviations

CAI: Chronic Ankle Instability
CAIT: Cumberland Ankle Instability Tool
FAAM-S: Foot and Ankle Ability Measure-Sport
HRQOL: Health-related Quality of Life
