# Supplementary material for: Prevalence and impact of chronic ankle instability in female sport: a cross-sectional study
Source: BMC Sports Sci Med Rehabil. 2025 Jul 8;17:183. doi: 10.1186/s13102-025-01211-5 (PMC12235940; doi:10.1186/s13102-025-01211-5)
Supplement: Supplementary file 1 — Supplementary Material 1 [file 13102_2025_1211_MOESM1_ESM.pdf]

Participant Information Sheet

. Thank you for your interest in completing our online survey.

This survey is live for you to complete at any time from 9th March 2024 until 22nd April 2024 (6 weeks).

The survey is completely anonymous. It includes questions about your sporting history, occupation, age, country you reside in, as well as your general health and ankle function.

Please note that it is your decision to take part in this investigation and you can exit the study survey at any point which will remove yourself from the investigation. All data collected is anonymised, so once you have submitted your survey, we will not be able to delete your submission. Not taking part in this study or withdrawal will not affect your standing or your relationship with any of the collaborating Universities in any way.

We expect the survey to take 20-25 minutes to complete.

Once submitted, the end of survey message will appear, and you may exit.

All survey data will be handled on Qualtrics software licensed to the University of Strathclyde. As data will be anonymous it will therefore be stored indefinitely.

The experiment offers neither incentives nor reimbursement.

Study Consent Form

Q2.1. ☐ I confirm that I have read and understood the Participant Information Sheet for the above project and the researcher has answered any queries to my satisfaction.
☐ I confirm that I have read and understood the Privacy Notice for Participants in Research Projects and understand how my personal information will be used and what will happen to it (i.e. how it will be stored and for how long).
☐ I understand that my participation is voluntary and that I am free to withdraw from the project at any time, up to the point of completion, without having to give a reason and without any consequences.
☐ I understand that I can request the withdrawal from the study of some personal information and that whenever possible researchers will comply with my request.

□ I understand that anonymised data (i.e. data that do not identify me personally) cannot be withdrawn once they have been included in the study.
□ I understand that any information recorded in the research will remain confidential and no information that identifies me will be made publicly available.
□ I consent to being a participant in the project.

○ I consent to being part of the study

○ I do not consent to being part of the study

Participant Information

Q3.1. This survey is assessing female athletes only. Are you female?

○ Yes

○ No

Q3.2. What sport do you play?

☐ Netball

☐ Soccer

☐ Basketball

☐ Volleyball

Q3.3. Have you played the above sport for at least 12 months?

- ○ Yes
- ○ No

Q3.4. How long have you played the above sport in total? Please answer in years.

[ ]

Q3.5. What age are you?

- ○ 18-24
- ○ 25-29
- ○ 30-39
- ○ 40-49
- ○ 50-59
- ○ 60-69
- ○ 70-79
- ○ 80-89
- ○ 90+

Q3.6. How tall are you? Please answer in cm.

Convert feet/inches to cm here: https://www.rapidtables.com/convert/length/feet-inch-to-cm.html?ft=5&inch=3

Q3.7. What do you weigh? Please answer in kg.

Convert lbs to kg here: https://www.rapidtables.com/convert/weight/pound to-kg.html

Q3.8. Where do you currently live?

- Australia
- United Kingdom
- United States of America
- New Zealand
- Other

Q3.9. What level of sport do you currently play?

- ☐ Professional
- ☐ International
- ☐ National
- ☐ Club level
- ☐ Recreational
- ☐ Social

Q3.10. What is the highest level of sport you have played?

- ☐ Professional
- ☐ International
- ☐ National
- ☐ Club level
- ☐ Recreational
- ☐ Social

Q3.11. If based in the United States, what is the highest level of sport you have played?

- ☐ National
- ☐ Professional
- ☐ Collegiate - NCAA Division I

- ☐ Collegiate - NCAA Division II
- ☐ Collegiate - NCAA Division III
- ☐ Collegiate - NAIA
- ☐ Collegiate - National Junior College Athletic Association
- ☐ University/College Club Level
- ☐ High School/Travel League
- ☐ Recreational/Social/Intramural

Q3.12. If based in the United States, what level of sport do you currently play?

- ☐ National
- ☐ Professional
- ☐ Collegiate - NCAA Division I
- ☐ Collegiate - NCAA Division II
- ☐ Collegiate - NCAA Division III
- ☐ Collegiate - NAIA
- ☐ Collegiate - National Junior College Athletic Association
- ☐ University/College Club Level
- ☐ High School/Travel League
- ☐ Recreational/Social/Intramural

Q3.13. The next few questions relate to ankle injuries.

Ankle sprains are the most common injury sustained by athletes and active individuals. In sports involving running,

quick stops and starts, cutting, and changing directions ankle sprains often occur. This can be from the ankle rolling when weight is planted on the outer edge of the foot or when an individual lands on uneven ground or another player's foot. There may be a popping sound when this happens as the ankle ligaments stretch/tear. The pain may be minimal and might quickly go away but in some cases the ankle may become swollen, painful to walk/run, and require medical treatment. In such cases crutches, walking boots, or braces may be prescribed for support and protection.

International Ankle Consortium definition of ankle sprain: *"An acute traumatic injury to the lateral ligament complex of the ankle joint as a result of excessive inversion of the rear foot or a combined plantar flexion and adduction of the foot. This usually results in some initial deficits of function and disability."*

International Ankle Consortium definition of giving way: *"The regular occurrence of uncontrolled and unpredictable episodes of excessive inversion of the rear foot (usually experienced during initial contact during walking or running), which do not result in an acute lateral ankle sprain."*

Q3.14. Have you ever had an ankle injury?

Your definition of an ankle sprain should be based on the above definition provided.

- ◯ Yes, a sprain to my left ankle
- ◯ Yes, a sprain to my right ankle
- ◯ Yes, I have sprained both ankles
- ◯ Yes, I have fractured my left ankle
- ◯ Yes, I have fractured my right ankle
- ◯ No, I have not injured my ankle

Q3.15. Based on the above definition of an ankle sprain, how many time have you sprained your left ankle?

____

Q3.16. Based on the above definition of an ankle sprain, how many time have you sprained your right ankle?

____

Ankle Function

. Please answer every question with one response that closely describes your condition within the past week. If the activity in question is limited by something other than your foot or ankle mark 'Not Applicable' (N/A)

. Because of your foot and ankle how much difficulty do you have with:

Q4.3. Running

- No difficulty
- Slight difficulty
- Moderate difficulty
- Extreme difficulty
- Unable to do
- N/A

Q4.4. Jumping

- No difficulty
- Slight difficulty
- Moderate difficulty
- Extreme difficulty

- Unable to do
- N/A

Q4.5. Landing

- No difficulty
- Slight difficulty
- Moderate difficulty
- Extreme difficulty
- Unable to do
- N/A

Q4.6. Starting and stopping quickly

- No difficulty
- Slight difficulty
- Moderate difficulty
- Extreme difficulty
- Unable to do
- N/A

Q4.7. Cutting/lateral movements

- No difficulty
- Slight difficulty

- Moderate difficulty
- Extreme difficulty
- Unable to do
- N/A

Q4.8. Ability to perform activity with your normal technique

- No difficulty
- Slight difficulty
- Moderate difficulty
- Extreme difficulty
- Unable to do
- N/A

Q4.9. Ability to participate in your desired sport as long as you like

- No difficulty
- Slight difficulty
- Moderate difficulty
- Extreme difficulty
- Unable to do
- N/A

Q4.10. How would you rate your current level of function during your sports related activities from 0 to 100 with 100 being your level of function prior to your foot or ankle problem and 0 being the inability to perform any of your usual daily activities?

Q4.11. Overall, how would you rate your current level of function?

- ○ Normal
- ○ Nearly normal
- ○ Abnormal
- ○ Severely abnormal

Ankle Stability

Q5.1. I have pain in my LEFT ankle

- ○ Never
- ○ During sport
- ○ Running on uneven surfaces
- ○ Running on level surfaces
- ○ Walking on uneven surfaces

- ○ Walking on level surfaces

Q5.2. I have pain in my RIGHT ankle

- ○ Never
- ○ During sport
- ○ Running on uneven surfaces
- ○ Running on level surfaces
- ○ Walking on uneven surfaces
- ○ Walking on level surfaces

Q5.3. My LEFT ankle feels UNSTABLE

- ○ Never
- ○ Sometimes during sport (not every time)
- ○ Frequently during sport (every time)
- ○ Sometimes during daily activity
- ○ Frequently during daily activity

Q5.4. My RIGHT ankle feels UNSTABLE

- ○ Never
- ○ Sometimes during sport (not every time)
- ○ Frequently during sport (every time)
- ○ Sometimes during daily activity

- ○ Frequently during daily activity

Q5.5. When I make SHARP turn, my LEFT ankle feels UNSTABLE

- ○ Never
- ○ Sometimes when running
- ○ Often when running
- ○ When walking

Q5.6. When I make SHARP turn, my RIGHT ankle feels UNSTABLE

- ○ Never
- ○ Sometimes when running
- ○ Often when running
- ○ When walking

Q5.7. When going down the stairs, my LEFT ankle feels UNSTABLE

- ○ Never
- ○ If I go fast
- ○ Occassionally
- ○ Always

Q5.8. When going down the stairs, my RIGHT ankle feels UNSTABLE

- ○ Never
- ○ If I go fast
- ○ Occassionally
- ○ Always

Q5.9. My LEFT ankle feels UNSTABLE when standing on ONE leg

- ○ Never
- ○ On the ball of my foot
- ○ With my foot flat

Q5.10. My RIGHT ankle feels UNSTABLE when standing on ONE leg

- ○ Never
- ○ On the ball of my foot
- ○ With my foot flat

Q5.11. My LEFT ankle feels UNSTABLE when

- Never
- I hop from side to side
- I hop on the spot
- When I jump

Q5.12. My RIGHT ankle feels UNSTABLE when

- Never
- I hop from side to side
- I hop on the spot
- When I jump

Q5.13. My LEFT ankle feels UNSTABLE when

- Never
- I run on uneven surfaces
- I jog on uneven surfaces
- I walk on uneven surfaces
- I walk on a flat surface

Q5.14. My RIGHT ankle feels UNSTABLE when

- Never

- I run on uneven surfaces
- I jog on uneven surfaces
- I walk on uneven surfaces
- I walk on a flat surface

Q5.15. TYPICALLY, when I start to roll over (or "twist") on my LEFT ankle, I can stop it

- Immediately
- Often
- Sometimes
- Never
- I have never rolled my ankle

Q5.16. TYPICALLY, when I start to roll over (or "twist") on my RIGHT ankle, I can stop it

- Immediately
- Often
- Sometimes
- Never
- I have never rolled my ankle

Q5.17. After a TYPICAL incident of my LEFT ankle rolling

over, my ankle returns to "normal"

- Almost immediately
- Less than one day
- 1-2 days
- More than 2 days
- I have never rolled over on my ankle

Q5.18. After a TYPICAL incident of my RIGHT ankle rolling over, my ankle returns to "normal"

- Almost immediately
- Less than one day
- 1-2 days
- More than 2 days
- I have never rolled over on my ankle

Quality of Life

Q6.1. Would you say that in general your health is:

- Excellent
- Very good
- Good
- Fair
- Poor

Q6.2. Now thinking about your physical health, which includes physical illness and injury, for how many days during the past 30 days was your physical health not good?

○ [ ] Number of days

○ None

Q6.3. Now thinking about your mental health, which includes stress, depression, and problems with emotions, for how many days during the past 30 days was your mental health not good?

○ [ ] Number of days

○ None

. If you answered “none” to questions 6.2 and 6.3, skip question 6.5 below:

Q6.5. During the past 30 days, for about how many days did poor physical or mental health keep you from doing your usual activities, such as self-care, work, or recreation?

- ◯ [ ] Number of days
- ◯ None

. Instructions: These next questions are about physical, mental, or emotional problems or limitations you may have in your daily life.

Q6.7. Are you LIMITED in any way in any activities because of any impairment or health problem?

- ◯ Yes
- ◯ No

Q6.8. What is the major impairment or health problem that limits your activities?

- ◯ Arthritis/rheumatism
- ◯ Back or neck problem

- ○ Fractures, bone/joint surgery
- ○ Walking problem
- ○ Lung/breathing problem
- ○ Hearing problem
- ○ Eye/vision problem
- ○ Heart problem
- ○ Stroke problem
- ○ Hypertension/high blood pressure
- ○ Diabetes
- ○ Cancer
- ○ Depression/anxiety/emotional problem
- ○ Other impairment/problem

Q6.9. For HOW LONG have your activities been limited because of your major impairment or health problem?

- ○ Days
- ○ Weeks
- ○ Months
- ○ Years

Q6.10. Because of any impairment or health problem, do you need the help of other persons with your PERSONAL CARE needs, such as eating, bathing, dressing, or getting around the house?

- ○ Yes

- [ ] No

Q6.11. Because of any impairment or health problem, do you need the help of other persons in handling your ROUTINE needs, such as everyday household chores, doing necessary business, shopping, or getting around for other purposes?

- [ ] Yes
- [ ] No

Q6.12. During the past 30 days, for about how many days did PAIN make it hard for you to do your usual activities, such as self-care, work, or recreation?

- [ ] ______ Number of days
- [ ] None

Q6.13. During the past 30 days, for about how many days have you felt SAD, BLUE, or DEPRESSED?

- [ ] ______ Number of days
- [ ] None

Q6.14. During the past 30 days, for about how many days have you felt WORRIED, TENSE, or ANXIOUS?

○ [ ] Number of days

○ None

Q6.15. During the past 30 days, for about how many days have you felt you did NOT get ENOUGH REST or SLEEP?

○ [ ] Number of days

○ None

Q6.16. During the past 30 days, for about how many days have you felt VERY HEALTHY AND FULL OF ENERGY?

○ [ ] Number of days

○ None

Fear of injury

Q7.1. I'm afraid I might injure myself if I exercise

- ◯ Strongly disagree
- ◯ Somewhat disagree
- ◯ Somewhat agree
- ◯ Strongly agree

Q7.2. If I were to try overcome it, my pain would increase

- ◯ Strongly disagree
- ◯ Somewhat disagree
- ◯ Somewhat agree
- ◯ Strongly agree

Q7.3. My body is telling me I have something dangerously wrong

- ◯ Strongly agree
- ◯ Somewhat agree
- ◯ Somewhat disagree
- ◯ Strongly disagree

Q7.4. My pain would probably be relieved if I were to exercise

- ○ Strongly agree
- ○ Somewhat agree
- ○ Somewhat disagree
- ○ Strongly disagree

Q7.5. People aren't taking my medical condition seriously enough

- ○ Strongly agree
- ○ Somewhat agree
- ○ Somewhat disagree
- ○ Strongly disagree

Q7.6. My accident has put my body at risk for the rest of my life

- ○ Strongly agree
- ○ Somewhat agree
- ○ Somewhat disagree
- ○ Strongly disagree

Q7.7. Pain always means I have injured my body

- ○ Strongly agree
- ○ Somewhat agree

- Somewhat disagree
- Strongly disagree

Q7.8. Just because something aggravates my pain does not mean it is dengerous

- Strongly agree
- Somewhat agree
- Somewhat disagree
- Strongly disagree

Q7.9. I am afraid that I might injure myself accidently

- Strongly agree
- Somewhat agree
- Somewhat disagree
- Strongly disagree

Q7.10. Simply being careful that I do not make any unnecessary movements is the safest think I can do to prevent my pain from worsening

- Strongly agree
- Somewhat agree
- Somewhat disagree

- Strongly disagree

Q7.11. I wouldn't have this much pain if there weren't something potentially dangerous going on in my body

- Strongly agree
- Somewhat agree
- Somewhat disagree
- Strongly disagree

Q7.12. Although my condition is painful, I would be better off if I were physically active

- Strongly agree
- Somewhat agree
- Somewhat disagree
- Strongly disagree

Q7.13. Pain lets me know when to stop exercising so that I don't injure

- Strongly agree
- Somewhat agree
- Somewhat disagree
- Strongly disagree

Q7.14. It's really not safe for a person with a condition like mine to be physically active

- Strongly agree
- Somewhat agree
- Somewhat disagree
- Strongly disagree

Q7.15. I can't do all the things normal people do because it's too easy for me to get injured

- Strongly agree
- Somewhat agree
- Somewhat disagree
- Strongly disagree

Powered by Qualtrics
